# Spiral-based 3D MR thermometry

**DOI:** 10.1186/2050-5736-3-S1-P18

**Published:** 2015-06-30

**Authors:** Samuel Fielden, Li Zhao, Wilson Miller, Xue Feng, Max Wintermark, Kim Butts Pauly, Craig Meyer

**Affiliations:** 1 University of Virginia, Charlottesville, Virginia, United States; 2 Stanford University, Stanford, California, United States

## Background/introduction

Real time MR thermometry, usually based on the proton-resonance frequency shift, is a key aspect of MR-guided focused ultrasound procedures. The desire to monitor the entire sonicated volume has led the field towards the development of rapid, 3D methods; however, acquiring fully sampled 3D volumetric data to monitor heating is time consuming, and so fast methods must be developed in order to meet the spatial and temporal requirements for adequate monitoring of thermal therapy. The data acquisition efficiency of spiral trajectories is higher than that of Cartesian scanning. Therefore, spiral trajectories are an attractive way to improve temporal resolution while maintaining spatial resolution in MR thermometry. We have recently reported that a variation on the traditional spiral-out trajectory, called the redundant spiral-in/out trajectory, has certain advantages in terms of off-resonance performance. We hypothesize this trajectory to also be advantageous for PRF thermometry. Here, we have implemented this –in/out trajectory, compared its performance in terms of the focal spot size and position shift *versus* a Cartesian and spiral-out acquisition, and have generated rapid 3D temperature maps using the method.

## Methods

All experiments were performed in a gel phantom, using an MR-compatible FUS system (RK-100, FUS Instruments Inc., Toronto) in a 3T whole-body scanner (Siemens Trio). 2D temperature maps were acquired with a GRE sequence with TR/TE = 15/6 ms, FA = 25-degrees, FOV = 64 mm2, matrix size 64 x 64. Spiral readout length was 1 ms. Spatial/temporal resolution for Cartesian imaging was 1x1x3 mm3/960 ms *vs*. 1x1x3 mm3/720 ms for spiral imaging. The size and relative shift of the ultrasound hot spot was measured for all acquisitions. For spiral 3D acquisitions, rapid imaging was achieved by a 3D interleaved stack-of-spirals spoiled GRE sequence. All 3D phase encoding partitions per volume were collected, for a total acquisition time per volume of 4.1 seconds. The redundant–in/out method requires 2X averaging, yielding a temporal footprint of 8.2 seconds per volume, reconstructed in a sliding window fashion. Sixteen 3D slices were acquired; all other MRI parameters were identical to the 2D experiments.

## Results and conclusions

**Figure 1:**
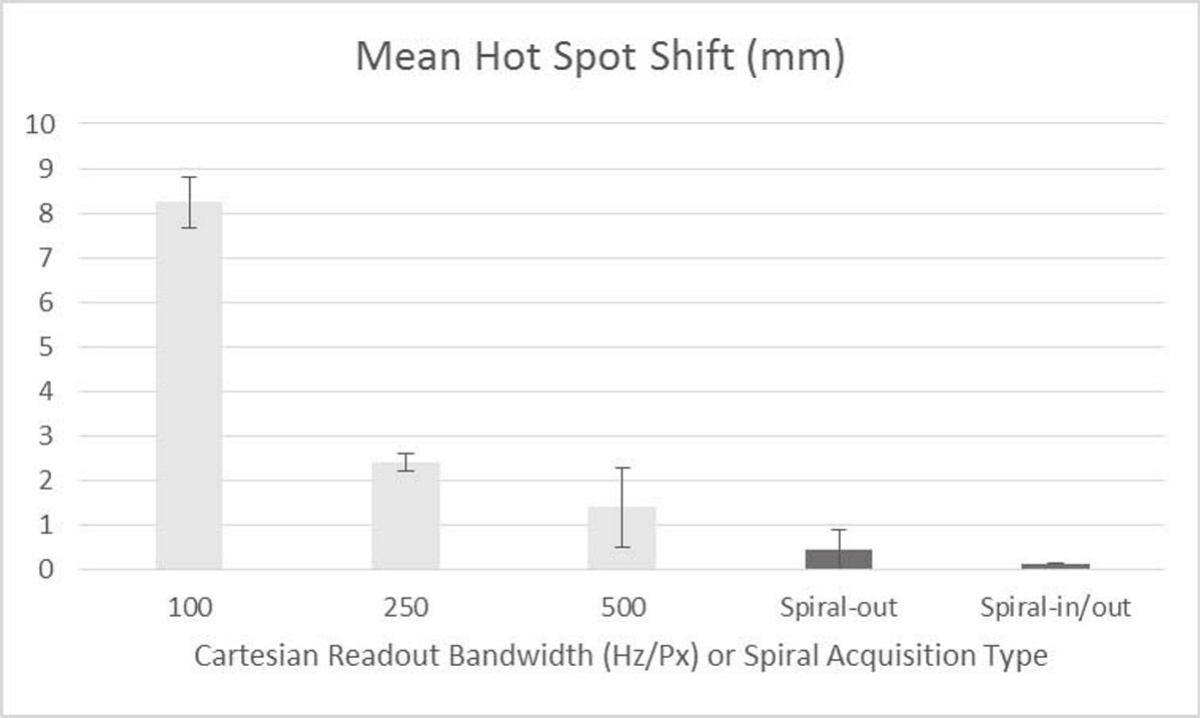
Focal spot shift measured between scans with switched readout / phase encoding directions. Low bandwidths are used to improve SNR in Cartesian imaging and in fast EPI methods, but result in greater shifts.

**Figure 2:**
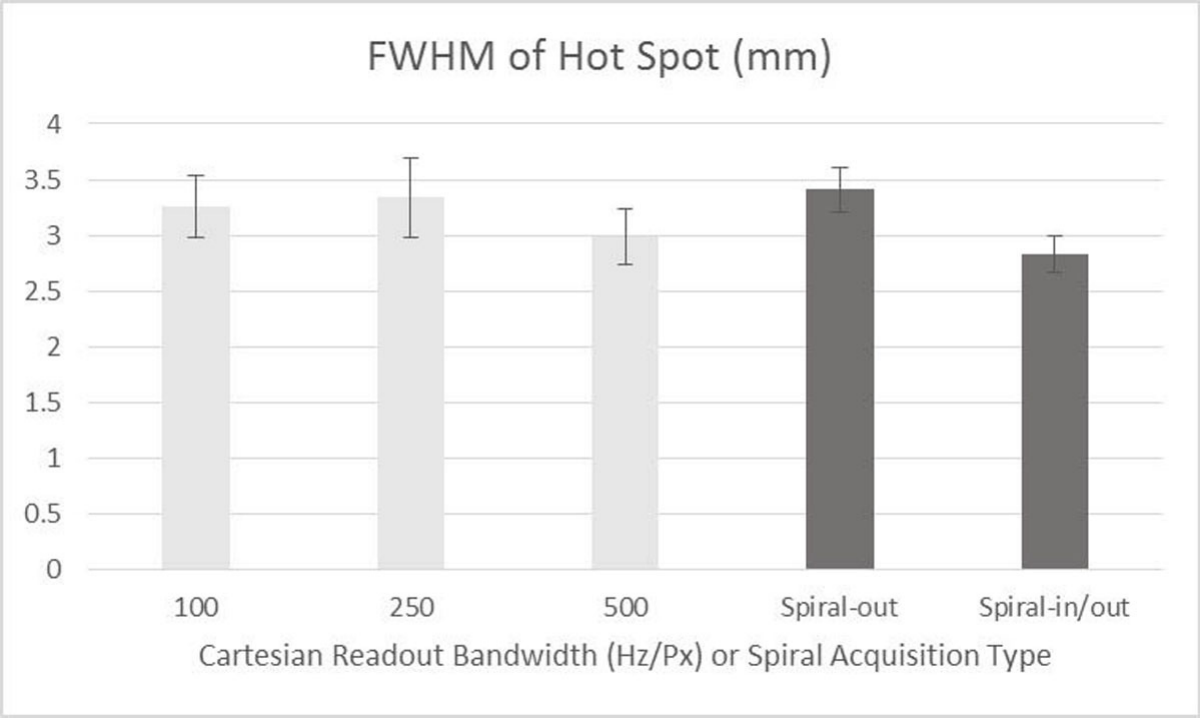
Full width at half maximum (FWHM) measurements of focal spot size at maximum temperature time point. Spiral-in/out recovers the slight blurring observed in the spiral-out images.

**Figure 3:**
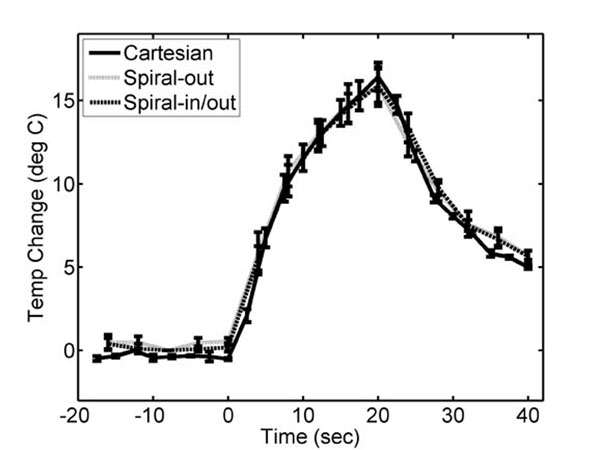
Measured temperature at ultrasound focal position for Cartesian, spiral-out, and spiral-in/out acquisitions.

**Figure 4:**
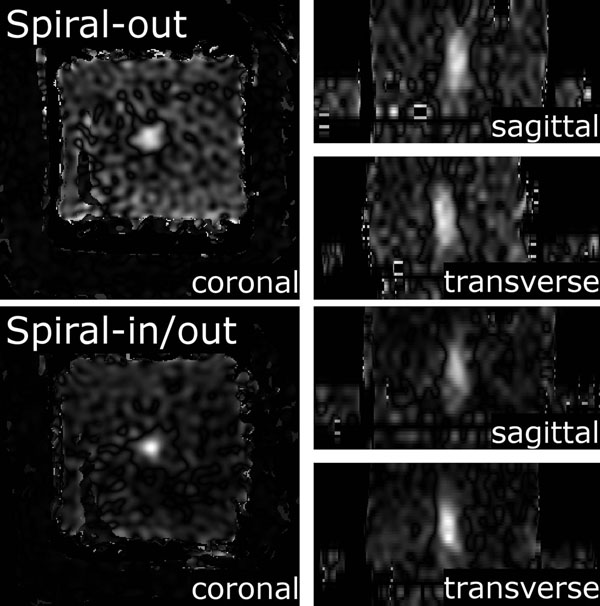
3D temperature map of phantom calculated from spiral-out data (top) and spiral- in/out data (bottom). The FUS focal spot is an ellipsoid with the long-axis oriented in the through-plane direction.

Figure 1 shows that, using spiral readouts, temperature-induced phase does not cause a position shift of the ultrasound focal spot. In Figure 2, we show that the hot spot is better resolved when using the redundant in/out method, compared to spiral-out. Figure 3 shows time-temperature plots of the hot spot, indicating the measured temperature using the spiral methods is accurate. Figure 4 shows 3D temperature maps obtained using the spiral-based methods. In conclusion, the efficiency of spiral readouts supports rapid generation of 3D temperature maps, with no shift of the focal spot. The reconstruction can easily be performed in real-time, with little concern for computational burden that exists for constrained methods.

